# Overlapping role of synaptophysin and synaptogyrin family proteins in determining the small size of synaptic vesicles

**DOI:** 10.1073/pnas.2409605121

**Published:** 2024-07-10

**Authors:** Daehun Park, Kenshiro Fujise, Yumei Wu, Rafael Luján, Sergio Del Olmo-Cabrera, John F. Wesseling, Pietro De Camilli

**Affiliations:** ^a^Department of Neuroscience, Yale University School of Medicine, New Haven, CT 06510; ^b^Department of Cell biology, Yale University School of Medicine, New Haven, CT 06510; ^c^Program in Cellular Neuroscience, Neurodegeneration and Repair, Yale University School of Medicine, New Haven, CT 06510; ^d^Department of Medical and Biological Sciences, The Catholic University of Korea, Bucheon 14662, South Korea; ^e^Department of Biotechnology, The Catholic University of Korea, Bucheon 14662, South Korea; ^f^Synaptic Structure Laboratory, Departamento de Ciencias Médicas, Instituto de Biomedicina de la Universidad de Castilla-La Mancha, Facultad de Medicina, University of Castilla-La Mancha, Albacete 02006, Spain; ^g^Institute for Neurosciences Consejo Superior de Investigaciones Científicas-Universidad Miguel Hernández, San Juan de Alicante 03550, Spain

**Keywords:** synaptic vesicle, synaptic vesicle biogenesis, synaptophysin, synaptogyrin

## Abstract

Members of the synaptophysin and synaptogyrin family are vesicle proteins with four transmembrane domains. In spite of their abundance in synaptic vesicle (SV) membranes, their role remains elusive and only mild defects at the cellular and organismal level are observed in mice lacking one or more family members. Here, we show that coexpression with synapsin in fibroblasts of each of the four brain-enriched members of this family—synaptophysin, synaptoporin, synaptogyrin 1, and synaptogyrin 3—is sufficient to generate clusters of small vesicles in the same size range of SVs. Moreover, mice lacking all these four proteins have larger SVs. We conclude that synaptophysin and synaptogyrin family proteins play an overlapping function in the biogenesis of SVs and in determining their small size.

## Results and Discussion

**Fig. 1.**
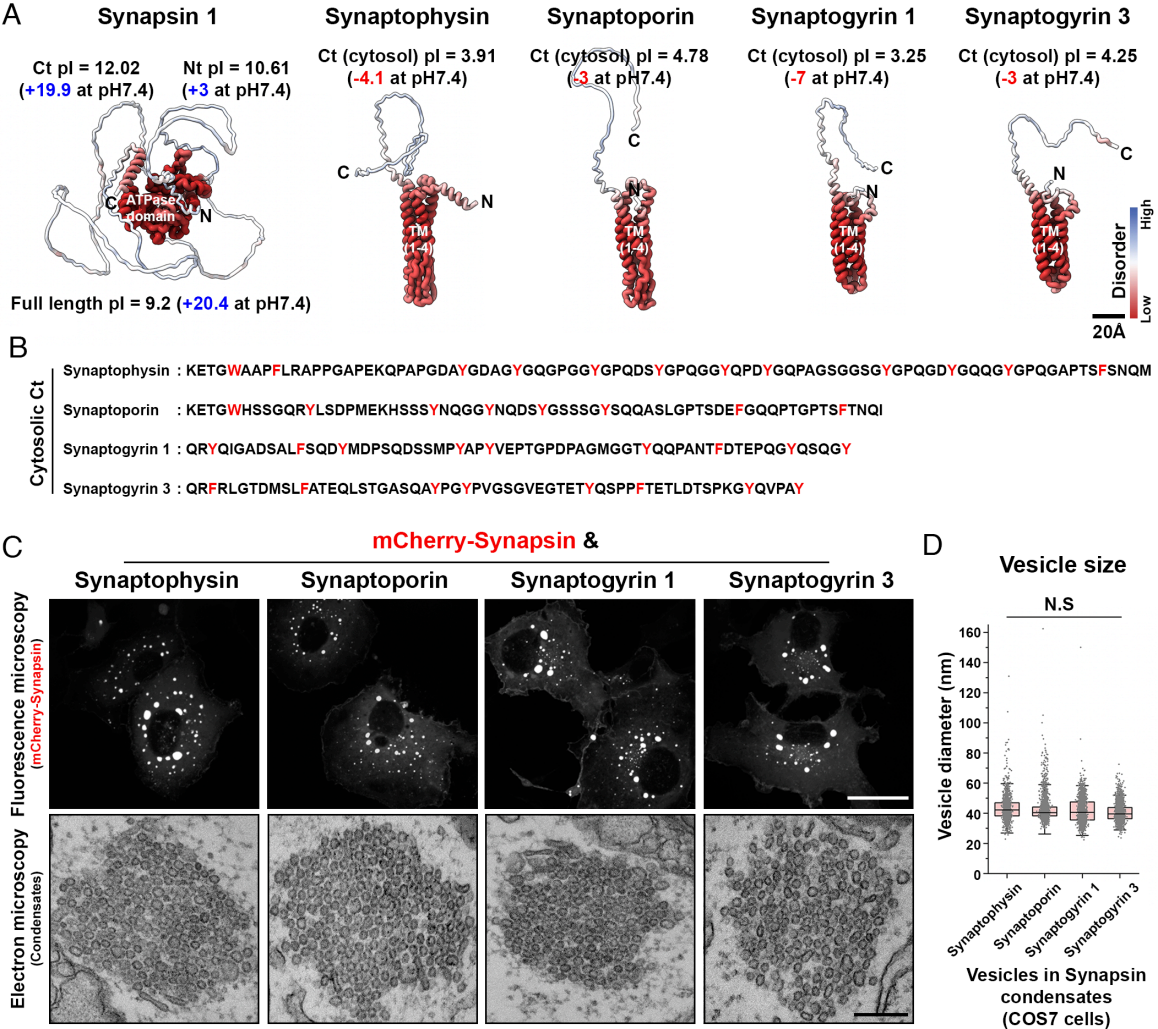
Synaptophysin and synaptogyrin family proteins form clusters of small vesicles with synapsin. (*A*) AlphaFold protein structures (human full length) and pI (isoelectric point) values of each protein. AFDB accession numbers: synapsin (AF-P17600-F1), synaptophysin (AF-P08247-F1), synaptoporin (AF-Q8TBG9-F1), synaptogyrin 1 (AF-O43759-F1), and synaptogyrin 3 (AF-O43761-F1). Colors represent disorderbility. The predicted protein structures were generated and visualized using AlphaFold (8) and Mol* Viewer (9). (*B*) Cytosolic C-terminal sequence of each protein. (*C*) Representative confocal (*Top*) and EM (*Bottom*) images of condensates from COS7 cells expressing as indicated. (*D*) Size distribution of the vesicles. Box plots show the median line (midline), 25/75 percentiles (boxes), and 2SD (whiskers). 1,469, 2,109, 1,978, and 2,887 vesicles were measured for synaptophysin, synaptoporin, synaptogyrin 1, and synaptogyrin 3 expressing cells, respectively (from four independent experiments). N.S; no significant difference by one-way ANOVA with Tukey’s HSD post hoc test. Scale bar: *A* = 20 Å, *C* = 20 µm (*Top*), 200 nm (*Bottom*).

A defining feature of synaptic vesicles (SVs) in nerve terminals is their very small size (1). However, the mechanisms accounting for such a characteristic remain poorly understood. We previously showed that ectopic expression in fibroblastic cells (COS7 cells) of synaptophysin, the second-most abundant integral membrane protein of SVs (2), along with synapsin, a peripheral SV protein which has the property to assemble into a macromolecular condensate (3), is sufficient to generate liquid clusters of small exo-endocytic recycling vesicles similar to SVs of synapses in size and reminiscent of such vesicles in molecular composition (4, 5). We further showed that the cytosolic C-terminal tail of synaptophysin, which is negatively charged [pI = 3.91 (−4.1 at pH 7.4)] and harbors tyrosine-containing repeats, mediates the interaction with the highly basic C-terminal tail of synapsin (4, 6) (Fig. 1 *A* and *B*). No such vesicle clusters were observed in fibroblasts when synapsin was expressed together with several other SV integral membrane proteins, including vesicle-associated membrane protein 2 (VAMP2), secretory carrier membrane 5 (SCAMP5), synaptotagmin 1, the vesicular glutamate transporter 1 (vGlut1), and the vesicular GABA transporter 1 (vGAT1), although these other proteins coassembled with synaptophysin if coexpressed with synaptophysin and synapsin (5). These results suggested a specialized role of synaptophysin in the generation of vesicles with the size range of SVs which can be clustered by synapsin. However, no obvious morphological and functional changes at synapses can be observed in synaptophysin knock-out (KO) mice (7).

Mice express three other SV proteins that share structural similarities with synaptophysin and are believed to have a common evolutionary origin (10): synaptoporin (synaptophysin 2), synaptogyrin 1, and synaptogyrin 3 (Fig. 1*A*). Not only do these proteins have the same overall structure and topology as synaptophysin (Fig. 1*A*)—they are members of the tetraspan vesicle membrane protein (TVP) family with short cytosolic domains—but also share in their short C-terminal cytosolic tail features that were shown to be important for the interaction between synaptophysin and synapsin (4): highly negative charge [4.78 (−3 at pH 7.4) for synaptoporin; 3.25 (−7 at pH 7.4) for synaptogyrin 1; 4.25 (−3 at pH 7.4) for synaptogyrin 3] (Fig. 1*A*) and abundant presence of aromatic amino acids (6) (Fig. 1*B*). Interestingly, we have now found that in contrast to what was observed for other SV proteins tested besides synaptophysin (5), expression in COS7 cells of each of these three other proteins together with synapsin resulted in the formation of clusters of small vesicles similar in size to those generated by synaptophysin and synapsin coexpression (Fig. 1 *C* and *D*). These results point to an overlapping role of these proteins in the biogenesis and clustering of SV.

**Fig. 2.**
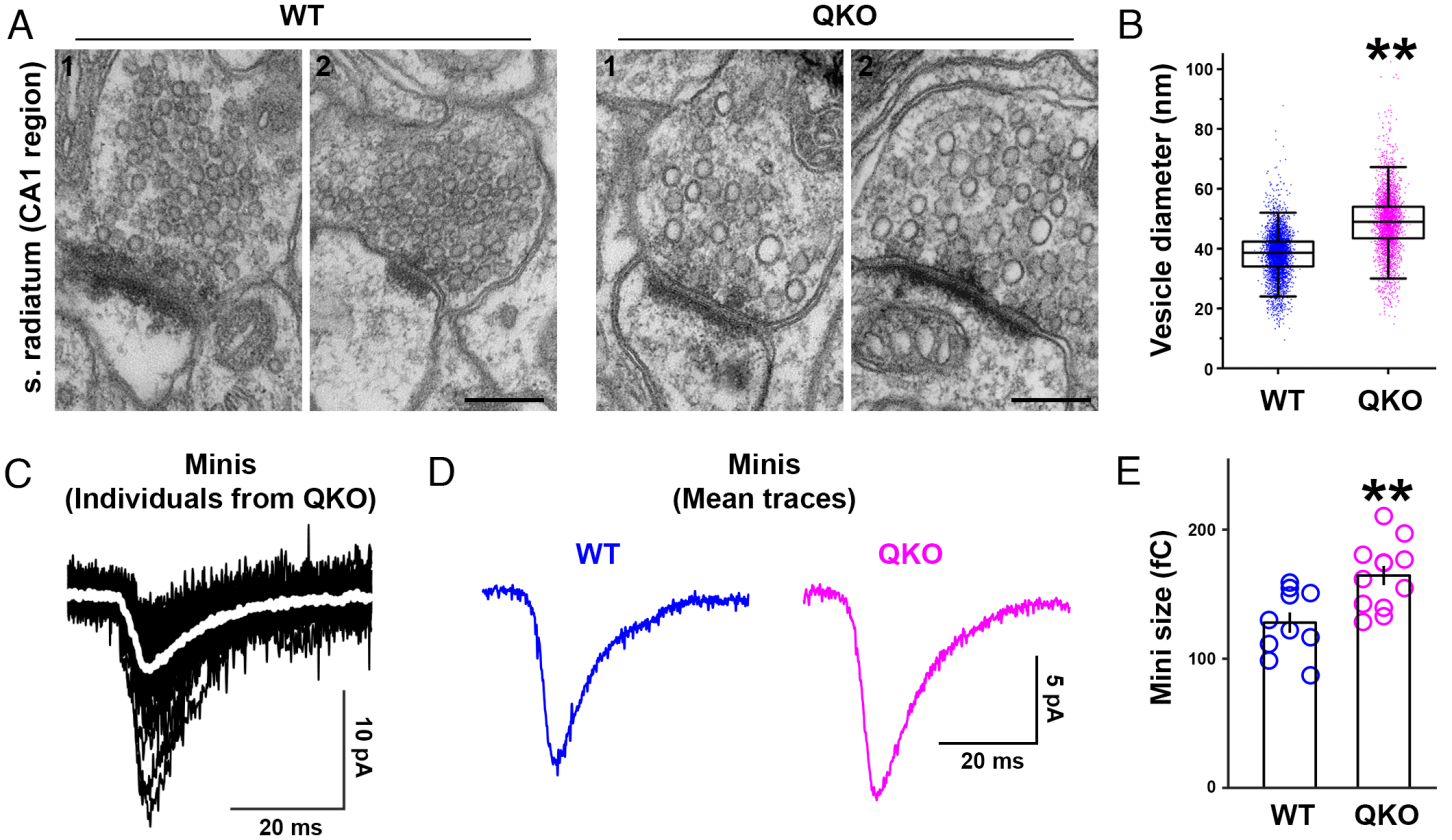
Increased size of SVs and electrophysiological quanta from synaptophysin, synaptoporin, synaptogyrin 1, and synaptogyrin 3 QKO mouse brains. (*A*) Representative EM micrographs from stratum radiatum (s. radiatum) in CA1 hippocampus of wild-type (WT) and QKO mutant mice. (*B*) Statistical analysis of (*A*). 5691 (WT) and 4099 (QKO) vesicles were measured (from four different tissue sections from two animals for each group). **P < 0.01 by Student’s *t* test. (*C–E*) Recordings were from CA1 pyramidal neurons, from 14- to 21-d-old mice. (*C*) Black traces are recordings of 32 minis from one QKO preparation. The superimposed white trace is the mean. (*D*) The mean of the means of all the minis detected for WT (blue) and QKO (magenta) neurons. (*E*) The current integrals of the mean mini for each neuron. **P < 0.01 by Student’s *t* test. (Scale bar: *A* = 200 nm.)

To validate this hypothesis, we examined by electron microscopy the morphology of synapses in the stratum radiatum of the CA1 region of the hippocampus from a previously generated mouse model in which all four synaptophysin/synaptogyrin family proteins had been knocked-out [quadruple knockout (QKO)]. These mice are viable and fertile, revealing that the four tetraspanins are not essential for synaptic transmission (11). However, they are prone to seizures, and studies of their synapses revealed increased neurotransmitter release in response to stimulation, consistent with subtle alterations in the neurotransmitter release machinery (11). Strikingly, we observed a clear increase in the average size of vesicles in presynaptic vesicle clusters relative to wild type [average diameter: 37.98 nm (WT) vs. 48.62 nm (QKO)] (Fig. 2 *A* and *B*), consistent with a role of synaptophysin/synaptogyrin family members in determining the small size of SVs (Fig. 1 *C* and *D*). Even if some of the larger vesicles represented endocytic intermediates rather than mature SVs, the difference reveals a defect in normal SV biogenesis.

Furthermore, spontaneous miniature synaptic currents (i.e., minis) were also increased by about 28% in QKO CA1 pyramidal neurons (Fig. 2 *C*–*E*), which matches the increase in mini size reported previously for calyx of Held synapses of QKO mice (11). Although the increase in mini size did not scale with the expected volume change (28% increase in diameter would imply a 110% increase in volume), it is consistent with the possibility that the individual vesicles contained more glutamate because of the larger size (Fig. 2).

We conclude that the four synaptophysin/synaptogyrin family proteins, while not required for the formation of neurotransmitter containing vesicles at synapses, may play a role in the acquisition of their characteristic very small size. Larger SVs had been observed at synapses of synaptogyrin null mutants of *Drosophila* that has a single synaptogyrin isoform and lacks a synaptophysin homolog (12). As we show here, synaptophysin/synaptogyrin family proteins are not only necessary to generate normal SVs but are also sufficient to generate small SV-like vesicles when expressed ectopically. A critical open question to be addressed is how these tetraspanins may determine vesicle shape and whether they do so by an intrinsic property or by recruiting to vesicles other factors with broad expression in neurons and nonneuronal cells.

## Materials and Methods

All animal experiments were approved by the corresponding Institutional Animal Care and Use Committee [IACUC of Yale University, and the Ethical Committee of the Generalitat Valenciana (2022/VSC/PEA/0255) for Alicante]. Mouse colony maintenance, cell culture, transfection, imaging, and statistical analyses were done as previously described (4, 5, 11, 13). All experiments and analysis comparing wild type and QKO were conducted blind to genotype. Additional details are provided in *SI Appendix*.

## Supplementary Material

Appendix 01 (PDF)

## Data Availability

All study data are included in the article and/or *SI Appendix*.
